# Incidence and prevalence of neurodevelopmental disorders and disabilities among métis children in Alberta, Canada: A retrospective birth cohort study

**DOI:** 10.1371/journal.pone.0333699

**Published:** 2025-10-03

**Authors:** Stuart Lau, Jesus Serrano-Lomelin, Matt Hicks, Reagan Bartel, Kelsey Bradburn, Ashton James, Susan Crawford, Jeffrey A. Bakal, Amy Colquhoun, Anne Hicks, Manoj Kumar, Rhonda J. Rosychuk, Alvaro Osornio-Vargas, Radha Chari, Maria B. Ospina

**Affiliations:** 1 Department of Obstetrics and Gynecology, University of Alberta, Edmonton, Alberta, Canada; 2 Department of Public Health Sciences, Queen’s University, Kingston, Ontario, Canada; 3 Department of Pediatrics, University of Alberta, Edmonton, Alberta, Canada; 4 Otipemisiwak Métis Government of the Métis Nation within Alberta, Edmonton, Alberta, Canada; 5 Alberta Health Services Provincial Research Data Services, Edmonton, Alberta, Canada; 6 Health Analytics, Alberta Health, Calgary, Alberta, Canada; Jawaharlal Institute of Postgraduate Medical Education and Research, INDIA

## Abstract

Limited research has examined the neurodevelopmental health of Métis children from a functional perspective, which is essential for culturally sensitive service planning, and policy development. This population-based retrospective birth cohort study linked provincial administrative health data of Métis and non-Métis singleton live births (2006–2016) to follow them up to 10 years of age. A random 1:4 sample of non-Métis children served as a reference group. Neurodevelopmental disorders and disabilities (NDD/D) were examined across six functional NDD/D domains. Prevalence odds ratios (pOR) with 95% confidence intervals (CI) were calculated using logistic regression models, adjusted for maternal and neonatal characteristics. Incidence rates (IR) per 1,000 person-years were estimated, and age-specific IR was modeled using longitudinal Poisson regression, adjusting for covariates. Associations between maternal and neonatal characteristics and NDD/D incidence among Métis children were examined using multivariable longitudinal Poisson regression models, with adjusted incidence rate ratios (IRR) and 95% CI reported. A total of 38,958 singleton live births were included (7,853 Métis and 31,105 non-Métis). Overall NDD/D prevalence among Métis (3.3%) and non-Métis (2.8%) children did not differ significantly after adjustment (adjusted pOR: 1.1, 95% CI: 0.9, 1.3). Learning-cognition was the most prevalent NDD/D domain. Métis children had a higher IR of NDD/D at age 2 (5.5 vs. 2.8 cases per 1,000 person-years, rate difference: 2.7 [95% CI: 0.8, 4.6]). Among Métis children, higher NDD/D incidence was associated with maternal age younger than 20 or older than 35 years, high pre-pregnancy weight, male sex, preterm birth, and congenital anomalies. While overall NDD/D prevalence was similar between Métis and non-Métis children, Métis children were more likely to be diagnosed at age 2, suggesting potential differences in early diagnosis, access to care, or underlying risk factors. A functional classification approach of neurodevelopmental health supports culturally responsive early screening and intervention strategies to address these differences.

## Introduction

Neurodevelopmental disorders and disabilities (NDD/D) encompass a range of conditions that affect core domains of child development, including motor, speech, cognitive, social, sensory, and neuropsychological functioning. Standard diagnostic systems such as the Diagnostic and Statistical Manual of Mental Disorders (DSM) [1], and the International classification of Diseases (ICD) [2] provide the criteria most commonly used to define and classify these conditions. At the same time, the term NDD/D is also used in research and policy contexts to describe functional profiles rather than categorical diagnoses, aligning with the International Classification of Functioning, Disability, and Health (ICF) [3], which is endorsed by the World Health Organization and adopted by many countries, including Canada. The ICF conceptualizes disability as an interaction between a health condition and environmental and personal factors, and categorizes disability based on impairments in body function and structure, activity limitations, and restrictions in social participation. This framework informs policy development, program design, and intervention planning, and its functional perspective has been highlighted in prior work discussing definitional and conceptual issues in studying disability [4]. Conditions commonly considered under NDD/D, whether defined through diagnostic systems (DSM, ICD) or described through functional profiles in the ICF framework include autism spectrum disorder (ASD), attention deficit-hyperactivity disorder (ADHD), specific learning disabilities, cerebral palsy (CP), and fetal alcohol spectrum disorder (FASD), among others. Although FASD is not separately classified as an NDD in DSM or ICD classifications, it is frequently recognized in Canadian clinical, administrative, and policy contexts as a neurodevelopmental disability due to its associated cognitive and learning difficulties. In Canada, the classification of NDD/D has been applied within provincial administrative health databases to identify children with these conditions [5,6].

Approximately 5% of Canadian children have a disability, with 74% of these cases attributed to an NDD/D [4]. These conditions often persist across the lifespan, contributing to social and mental health challenges, as well as reduced future employment and economic opportunities [7,8]. Research on NDD/Ds among Indigenous children in Canada has largely taken a pan-Indigenous approach by aggregating data from First Nations, Métis, and Inuit populations –the three constitutionally recognized Indigenous groups in Canada– or by focusing exclusively on First Nations children. Most studies have examined older age groups, including adolescents and adults (15 years and older) [9–13]. Existing evidence suggests that Indigenous children have higher prevalence of developmental and learning disabilities, as well as ADHD symptoms, compared to non-Indigenous children [9,10], while ASD appears to be less prevalent [11,14]. Additionally, Indigenous children with CP experience worse health outcomes, including higher rates of injury and associated impairments compared to their non-Indigenous peers [12].

The prevalence of NDD/D among Indigenous children in Canada is influenced by the enduring impacts of colonialism, including intergenerational trauma, systemic racism, and discrimination. Historical factors such as forced displacement, residential schools, and cultural genocide have had significant implications for Indigenous child development [15]. Structural inequities contribute to increased exposure to adverse social determinants of health and barriers to timely, culturally appropriate healthcare [16]. These barriers are particularly evident in the diagnosis and support of neurodevelopmental conditions, with Métis children often being overlooked in research and healthcare policies [17]. A systematic review identified a lack of Métis-specific data on NDD/D prevalence and incidence, limiting the ability to develop tailored interventions [18]. This is a critical gap in research specific to the neurodevelopmental health of Métis children. In collaboration with the Otipemisiwak Métis Government of the Métis Nation within Alberta (MNA), this study evaluates the prevalence and incidence of NDD/D among Métis children in Alberta, with a group of non-Métis children serving as a reference group. Additionally, the study explores the association between maternal and neonatal characteristics and NDD/D incidence among Métis children. By addressing these gaps, this research aims to contribute to a more inclusive understanding of neurodevelopmental health disparities in Canada.

## Materials and methods

### Study design and setting

This population-based retrospective birth cohort study used de-identified administrative health data from Alberta. Alberta, a province in western Canada with a population of approximately 4.8 million people, is home to 284,465 Indigenous people, representing 6.8% of the total population. Among them, approximately 127,475 identify as Métis, making it the second-largest Métis population in Canada [19]. Indigenous children under 14 years comprise 25% of the Indigenous population in Canada and 7% of all children, with an estimated 160,000 Métis children across the country [20].

### Data sources and linkage procedures

The study used de-identified, individual-level, longitudinal administrative health data from 2006 to 2019. It linked administrative health data from the Ehawawisit study [21], a retrospective birth cohort examining maternal and perinatal health among Métis people in Alberta. This analysis expands the original study by incorporating longitudinal administrative health data on children born from singleton pregnancies within the cohort. The Alberta Health Analytics and Performance Reporting Branch, the Alberta Perinatal Health Program (APHP), and the Alberta Health Services Provincial Research Data Services facilitated linkage of the Ehawawisit cohort data with these extended datasets.

Details on the creation of the Ehawawisit dataset has been reported elsewhere [21]; briefly, it was developed through probabilistic linkage of the MNA Identification Registry with the Alberta provincial population registry followed by deterministic matching with the APHP perinatal registry using personal health numbers. The MNA Identification Registry contains demographic data on registered Métis citizens [22], representing approximately 35% of Métis individuals in Alberta at the time of the Ehawawisit study [23]. The Alberta Health Care Insurance Plan (AHCIP) provides information registration on all Alberta residents. The APHP registry is a validated perinatal database with maternal and neonatal data for all hospital deliveries and midwife-attended home births in the province. The APHP includes linked maternal-child records, enabling longitudinal follow-up of child health outcomes.

For longitudinal follow-up of NDD/D outcomes, additional administrative health data were deterministically linked using personal health numbers. The Discharge Abstracts Database (DAD) captures hospitalization data, including diagnostic and procedural codes based on the International Classification of Diseases (ICD), 10^th^ Revision, enhanced Canadian Version (ICD-10-CA) [24] and the Canadian Classification of Health Interventions (CCI) [25]. The Alberta Physician Claims Assessment (APCA) database records services provided by fee-for-service physicians, using International Classification of Diseases, Ninth Revision (ICD-9) diagnostic codes [26] and CCI procedural codes. The National Ambulatory Care Reporting System (NACRS) collects data on emergency department visits and outpatient services, coded with ICD-10-CA and CCI. The Alberta Vital Statistics Birth and Deaths file maintains records of all births, deaths, marriages, and stillbirths in the province. Daya linkage for the study was completed and released to investigators on August 23, 2021.

### Study population

The study included all singleton births to Métis mothers in Alberta between April 1, 2006 and March 31^st^, 2016, as identified in the Ehawawisit study [21]. A randomly selected 1:4 comparison group of singleton births to non-Métis mothers was identified using a probabilistic approach. The representativeness of the non-Métis sample was assessed by comparing maternal demographic and clinical characteristics, including age at delivery, mode of delivery, type of labour, area of residence, and socioeconomic status. The study cohorts were then linked to Alberta’s administrative health data to assess children’s NDD/D outcomes during the first ten years of life.

Each child in the study cohorts was observed from birth until one of the following events: reaching 10 years of age, the end of the study period in 2019, or death/migration out of the province. As a result, follow-up time varied across individuals; for example, children born between 2006 and 2009 contributed up to 10 years of observation, while those born in 2016 contributed up to 3 years.

### Identification of NDD/D cases

To identify NDD/D cases in the study population, we followed a functional approach used by Arim et al. [6], which applies ICD-9/ICD-10 diagnostic codes from the Participation and Activity Limitation Survey (PALS) [4] to classify 23 NDD/D conditions into six functional limitation domains: motor functioning, speech/communication, learning-cognition, reciprocal social interaction, sensory impairments, and neuropsychological. A child was classified as having an NDD/D if they had at least two ambulatory medical encounters or one hospitalization with a diagnostic code for an NDD/D condition [6] during the observation period. When a case was identified through two physician claims, the diagnosis date was assigned to the second claim, at which point the case was considered valid [5]. The index date was defined as the date of the first recorded diagnosis for any of the 23 NDD/D conditions. Supplementary S1 Table provides the full list of diagnostic codes and their corresponding functional domains. Conditions with no recorded cases were excluded from the analysis.

### Study covariates

Maternal sociodemographic and clinical characteristics, along with neonatal factors were included as covariates. These variables were selected based on their well-documented association with adverse child health outcomes and their theoretical relevance [27–33]. Maternal age at delivery was categorized in three groups (<20, 20–34, ≥ 35 years) to reflect differences in obstetric and health risks across age ranges [34]. Other maternal covariates obtained from the APHP included, high pre- pregnancy weight (defined as ≥91 kg in the APHP registry), smoking, and substance use, including alcohol use during pregnancy (≥3 drinks on any occasion during pregnancy or ≥1 drink per day throughout pregnancy), presence of a pre-existing medical condition (at least one of diabetes mellitus, heart disease, hypertension, renal disease), presence of a pregnancy-related condition (at least one of gestational hypertension, gestational diabetes, preeclampsia, eclampsia, placenta previa, hemorrhage, premature rupture of membranes), prenatal care (adequate, intermediate, intensive, inadequate, no care) [35], delivery mode (spontaneous vaginal, vacuum/forceps-assisted, or caesarean), and area of residence, classified as urban/metropolitan centre or rural/small population centre based on population density and proximity to urban centres [36]. Socioeconomic status (SES) was assessed using the Pampalon Material and Social Deprivation Index [37], derived from Canadian census data (2006, 2016). This index includes two components: material deprivation (based on income, education level, and employment), and social deprivation (based on marital status, one-person households, and single-parent families). The index is reported in quintiles, with Q_1_ representing the least deprived areas and Q_5_ the most deprived. Maternal postal codes at delivery were linked to the 2006 census for births between 2006 and 2010, and the 2016 census for births from 2011 to 2016.

Neonatal covariates included infant sex, presence of any congenital anomaly, preterm birth (PTB; < 37 weeks gestation), small-for-gestational age (SGA; birth weight < 10^th^ percentile), and large for gestational age (LGA; birth weight > 90^th^ percentile), based on gestational age-specific infant birth weight reference values from a population-based Canadian reference [38].

### Statistical analysis

Maternal sociodemographic, clinical, and neonatal characteristics of Métis and non-Métis children were summarized using frequencies and percentages. Missing values were not imputed. The number of missing values for each variable was reported, and data for variables with 20 or fewer observations were suppressed as per privacy guidelines [39]. Chi-square tests were used to compare the distribution of maternal and neonatal characteristics between Métis and non-Métis children, with statistical significance set at p < 0.05.

The period prevalence of NDD/D and domain-specific NDD/D (2006–2019) was calculated as the number of children meeting the criteria for at least one NDD/D or a diagnosis within a specific NDD/D domain, divided by all singleton live births between 2006 and 2016. Prevalence estimates were expressed as percentages with 95% confidence intervals (CI), calculated using the Agresti-Coull method [40]. Logistic regression models were used to estimate crude and adjusted prevalence odds ratios (pOR) with 95% CI, comparing overall NDD/D and domain-specific NDD/D conditions between Métis and non-Métis children. The overall NDD/D pOR was adjusted for maternal age, area of residence, material and social deprivation, smoking/substance use during pregnancy (combined), having at least one pre-existing medical condition/high pre-pregnancy weigh (combined), having at least one pregnancy-related condition, prenatal care, delivery mode, infant sex, PTB, SGA (non-PTB), LGA, and any congenital anomaly. To minimize multicollinearity, correlated covariates were combined when appropriate (e.g., smoking was combined with substance use during pregnancy, and any pre-existing medical condition with high pre-pregnancy weigh). Due to the lower frequency of domain-specific NDD/D conditions, domain-specific pOR were adjusted for maternal age and area of residence only.

The incidence rate of NDD/D for the period between 2006 and 2019 was calculated separately for Métis and non-Métis children as the total number of new NDD/D cases identified during this period, divided by the total person-time at risk. Person-time was defined as the cumulative time each child remained free from any NDD/D condition until diagnosis, death, or the end of the study period; whichever occurred first.

Age-specific incidence rates of NDD/D were calculated for Métis and non-Métis children, with each year of life (ages 1–10) treated as a distinct age group. The numerator was the number of new NDD/D cases within each age group, while the denominator was the total person-time at risk, defined as the cumulative time each child remained free from a specific NDD/D condition until they moved out of the province, died, turned 10 years old, or reached the end of the study period, whichever occurred first. Predicted age-specific incidence rates were estimated using a longitudinal Poisson regression model adjusted for covariates to ensure comparability between groups. In this model, follow-up time was included as an offset variable, and age was nested within individuals. Covariates for adjustment included maternal age, smoking/substance use during pregnancy, material and social deprivation, area of residence, at least one pre-existing medical condition/high pre-pregnancy weight, at least one pregnancy-related condition, delivery mode, prenatal care, infant sex, PTB, SGA (non-PTB), LGA, and any congenital anomaly. The difference in predicted incidence rates between Métis and non-Métis children at each age was assessed using post-estimation tests and expressed as rate differences. We reported the adjusted predicted incidence rates and rate differences with 95% CI. All incidence rates were expressed as the number of new NDD/D cases per 1,000 person-years.

Associations between maternal and neonatal characteristics and the incidence of NDD/D were estimated separately for Métis and non-Métis children using multivariable longitudinal Poisson regression models. Estimates for non-Métis children were included for comparative purposes. The characteristics examined included maternal age groups, material and social deprivation quintiles, area of residence, high maternal pre-pregnancy weight, smoking/substance use during pregnancy, at least one pre-existing medical condition, at least one pregnancy-related condition, delivery mode, prenatal care, infant sex, PTB, SGA (non-PTB), LGA, and any congenital anomaly. Crude and adjusted incidence rate ratios (IRR) with 95% CI were reported. Statistical analyses were conducted using STATA (Release 16. StataCorp LLC, College Station, TX).

### Ethics statement

Study variables were selected in collaboration with the MNA to ensure relevance for policies aimed at improving the well-being of Métis children. Ethics approval was obtained from the University of Alberta’s Health Research Ethics Board (#Pro00098620) and Queen’s University Health Research Ethics Board (TRAQ# 6037209). Because the study involved the secondary use of de-identified administrative health data, individual patient consent was not required and the authors did not access information that could identify individual participants during or after data collection. The study adhered to the REporting of studies Conducted using Observational Routinely-collected health Data (RECORD) guidelines for observational epidemiological studies [41], the CONSolIDated critERia for strengthening the reporting of health research involving Indigenous Peoples (CONSIDER) statement [42], and the six princples of ethical Métis research (reciprocal relationships, respect, safe and inclusive environments, diversity, Métis relevance, and context considerations) [43]. Additional information regarding the ethical, cultural, and scientific considerations specific to inclusivity in global research is included in the supporting information (S4 File).

## Results

**Fig 1:**
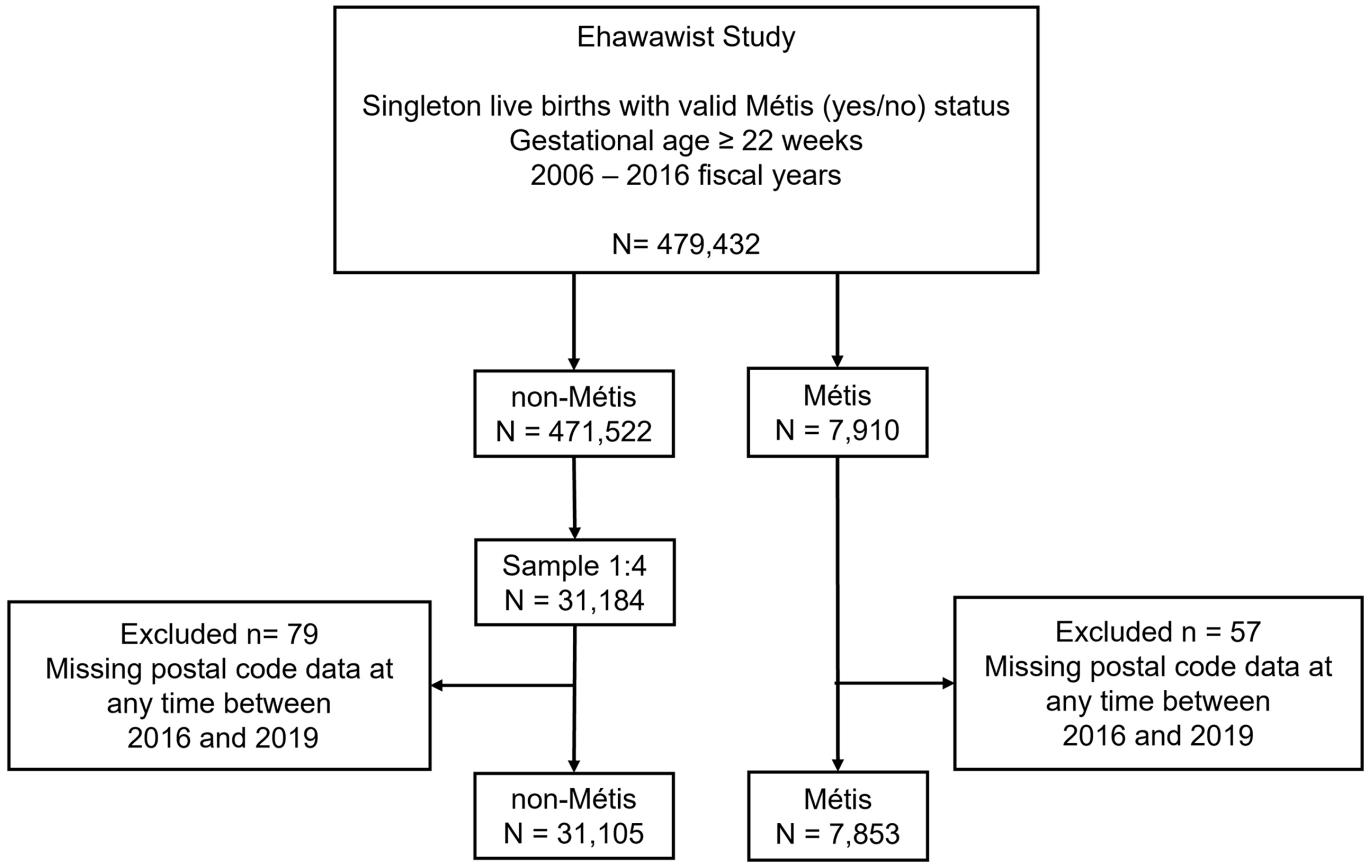
Study cohort flow diagram. APHP = Alberta Perinatal Health Program, MNAIR = Métis Nation of Alberta Identification Registry; AHCIP = Alberta Health Care Insurance Plan.

A total of 38,958 singleton live births were included in the study, comprising 7,853 children born to Métis mothers and 31,105 in the reference sample of children born to non-Métis mothers (**Fig 1**).

Table 1 presents the maternal sociodemographic, clinical, and neonatal characteristics of singleton live births to Métis and non-Métis women in the study population. Live births to Métis women were more likely to occur among younger mothers, with 9.5% of Métis live births occurring in mothers under 20 years of age, compared to 4% among live births to non-Métis women. A higher proportion of births to Métis women occurred in rural or small population centres (47.4% vs. 32% in non-Métis). Métis live births occurred more frequently in the most materially deprived areas (50.6% vs. 41.7%) and the most socially deprived areas (49.1% vs. 42%). Live births to Métis women were more frequently associated with maternal smoking during pregnancy (30.5% vs. 13.9%), and substance use during pregnancy (6.1% vs. 2.9%). High maternal pre-pregnancy weight (14.3% vs. 9.1%) and pre-existing medical conditions (11% vs. 10%) were more common in Métis live births while pregnancy-related morbidity was similar between the two groups. Patterns of prenatal care were largely comparable between the two groups. Most live births were associated with intermediate prenatal care, though a smaller proportion of Métis live births received no prenatal care compared to non-Métis live births (3.5% vs. 4.9%). Spontaneous/vaginal delivery was more common among Métis live births (64.9% vs. 59.8% in non-Métis), while caesarean deliveries were slightly less frequent (25.7% vs. 27.9%). Regarding neonatal characteristics, Métis infants were less likely to be born SGA (7% vs. 9.2% in non-Métis), while LGA births were more frequent among Métis infants (12.7% vs. 9.2%). The percentages of congenital anomalies, and PTB were comparable between groups.

**Table 1:** Maternal sociodemographic, clinical and neonatal characteristics of Métis and non-Métis singleton live births in the study population.

Characteristic	Métis N = 7,853 n (%)	Non-Métis N = 31,105 n (%)
**Sociodemographic**		
Maternal age group (years)^a^		
< 20	748 (9.5)	1,237 (4.0)
20-34	6,179 (78.7)	23,110 (74.3)
≥ 35	926 (11.8)	6,758 (21.7)
Area of residence at delivery^a^		
Rural or small population centre	3,723 (47.4)	9,950 (32.0)
Urban or metropolitan centre	4,056 (51.7)	20,953 (67.4)
Unclassified	74 (0.9)	202 (0.6)
Material deprivation quintile^a^		
Q_1_ (least deprived)	845 (10.8)	5,319 (17.1)
Q_2_	1,173 (14.9)	5,509 (17.7)
Q_3_	1,458 (18.6)	5,826 (18.7)
Q_4_	1,740 (22.2)	5,876 (18.9)
Q_5_ (most deprived)	2,233 (28.4)	7,085 (22.8)
Unclassified	404 (5.1)	1,490 (4.8)
Social deprivation quintile^a^		
Q_1_ (least deprived)	892 (11.4)	4,679 (15.0)
Q_2_	1,139 (14.5)	5,611 (18.4)
Q_3_	1,560 (19.9)	6,237 (20.1)
Q_4_	1,972 (25.1)	6,605 (21.2)
Q_5_ (most deprived)	1,886 (24.0)	6,483 (20.8)
Unclassified	404 (5.1)	1,490 (4.8)
**Maternal clinical factors**		
High pre-pregnancy weight (≥91 kg)^a^		
Yes	1,119 (14.3)	2,284 (9.1)
No	6,662 (84.8)	28,078 (90.3)
Missing	72 (0.9)	203 (0.6)
Smoke during pregnancy^a^		
Yes	2,392 (30.5)	4,309 (13.9)
No	5,389 (68.6)	26,593 (85.5)
Missing	72 (0.9)	203 (0.6)
Substance use during pregnancy^a^		
Yes	480 (6.1)	888 (2.9)
No	7,301 (93.0)	30,014 (96.5)
Missing	72 (0.9)	203 (0.6)
At least one pre-existing medical condition^a^		
Yes	865 (11.0)	3,096 (10.0)
No	6,988 (89.0)	28,009 (90.0)
At least one pregnancy-related conditions		
Yes	1,730 (22.0)	7,004 (22.5)
No	6,123 (78.0)	24,101 (77.5)
Prenatal care^a^		
Adequate	1,626 (20.7)	6,383 (20.5)
Intermediate	4,366 (55.6)	17,148 (55.1)
Intensive	146 (1.9)	473 (1.5)
Inadequate	1,397 (17.8)	5,431 (17.5)
No care	278 (3.5)	1,528 (4.9)
Missing	40 (0.5)	142 (0.5)
Delivery mode^a^		
Spontaneous vaginal	5,097 (64.9)	18,611 (59.8)
Vacuum/Forceps	717 (9.1)	3,741 (12.0)
Cesarean	2,015 (25.7)	8,682 (27.9)
Missing	24 (0.3)	71 (0.2)
**Neonatal factors**		
Sex at birth		
Female	3,931 (50.1)	15,246 (49.0)
Male	3,922 (49.9)	15,859 (51.0)
Congenital anomaly^a^		
Yes	136 (1.7)	397 (1.3)
No	7,645 (97.4)	30,506 (98.1)
Missing	72 (0.9)	202 (0.6)
Preterm birth		
Yes	567 (7.2)	2,186 (7.0)
No	7,286 (92.8)	28,919 (93.0)
Small for gestational age^**a**^		
Yes	550 (7.0)	2,870 (9.2)
No	7,287 (92.8)	28,198 (90.7)
Missing	16 (0.2)	37 (0.1)
Large for gestational age^a^		
Yes	997 (12.7)	2,873 (9.2)
No	6,840 (87.1)	28,195 (90.6)
Missing	16 (0.2)	37 (0.1)

^a^ Statistically significant difference (p < 0.05) between Métis and non-Métis groups based on Chi-square test.

### Period Prevalence of NDD/D diagnoses among Métis and non-Métis Children

A total of 1,116 children were diagnosed with at least one NDD/D condition, including 259 Métis and 857 non-Métis children. Among children diagnosed with NDD/D, most had a single diagnosis, with 90% (n = 233) of diagnosed Métis children and 88.5% (n = 758) of diagnosed non-Métis children having only one NDD/D diagnosis. The remaining 10% of diagnosed Métis children (n = 26) and 11.5% of diagnosed non-Métis children (n = 99) received multiple NDD/D diagnoses during the study period.

Table 2 presents the period prevalence of NDD/D diagnoses, including domain-specific prevalence, along with crude and adjusted pOR estimates comparing Métis and non-Métis children. The overall prevalence of NDD/D was 3.3% (95% CI: 2.9, 3.7) in Métis children and 2.8% (95% CI: 2.6, 2.9) in non-Métis children, corresponding to a crude pOR of 1.2 (95% CI: 1.1, 1.4). However, after adjusting for maternal, sociodemographic, clinical, and neonatal covariates, the association was no longer statistically significant (adjusted pOR: 1.1, 95% CI: 0.9, 1.3).

**Table 2:** Period prevalence of overall and domain-specific NDD/D diagnoses among Métis and non-Métis children, with crude and adjusted prevalence odds ratios.

	Métis		Non-Métis			
Domain	n	Prevalence % (95% CI)	n	Prevalence % (95% CI)	Crude pOR (95% CI)	apOR^b^ (95% CI)
NDD/D (All)	259^a^	3.3 (2.9, 3.7)	857^**a**^	2.8 (2.6, 2.9)	1.2 (1.1, 1.4)	1.1 (0.9, 1.3)
Motor functioning	<20^c^	0.2 (0.1, 0.4)	55	0.2 (0.1, 0.2)	1.2 (0.7, 2.1)	1.3 (0.7, 2.2)
Speech/ communication	55	0.7 (0.5, 0.9)	207	0.7 (0.6, 0.8)	1.1 (0.8, 1.4)	1.2 (0.9, 1.6)
Learning cognition	143	1.8 (1.6, 2.1)	424	1.4 (1.2, 1.5)	1.3 (1.1, 1.6)	1.2 (1.0, 1.5)
Reciprocal social interaction	26	0.3 (0.2, 0.5)	140	0.5 (0.4, 0.5)	0.7 (0.5, 1.1)	0.8 (0.5, 1.2)
Sensory impairments	<20^c^	0.2 (0.1, 0.3)	35	0.1 (0.1, 0.2)	1.6 (0.9, 3.0)	1.6 (0.8, 2.9)
Neuro- psychological	30	0.4 (0.3, 0.6)	91	0.3 (0.2, 0.4)	1.3 (0.9, 2.0)	1.4 (0.9, 2.1)

NDD/D = Neurodevelopmental disorders and disabilities; CI = confidence interval; pOR = prevalence odds ratio; apOR = adjusted pOR.

^a^ The NDD/D (All) n is lower than the sum of domain cases because some children were diagnosed in multiple domains.

^b^ NDD/D (All) pOR was adjusted for maternal age, area of residence, material and social deprivation, smoking/substance use during pregnancy, at least one pre-existing medical condition/high pre-pregnancy weight, at least one pregnancy-related condition, prenatal care, delivery mode, infant sex, preterm birth, small for gestational age (non-preterm), large for gestational age, and any congenital anomaly. Domain-specific pOR were only adjusted for maternal age and area of residence due to the lower frequency of domain-specific NDD/D conditions.

^c^ Exact numbers for variables with fewer than 20 observations are suppressed

Among NDD/D domains, the learning–cognition domain had the highest prevalence in both groups (Table 2). The crude pOR of 1.3 (95% CI: 1.1, 1.6) indicated higher odds of a learning–cognition NDD/D diagnosis among Métis children; however, this association was no longer statistically significant after adjusting for covariates (adjusted pOR: 1.2%; 95% 1.0, 1.5). The prevalence of all other NDD/D domains remained below 1% in both groups. While minor differences were observed across domains, none of the associations remained statistically significant after covariate adjustment.

### Incidence rates of NDD/D in Métis and non-Métis children

More than half of children (52.2%) were followed until 8–10 years of age. Approximately one-third (32.9%) were followed until they reached 10 years of age, while 5% had follow-up limited to 3 years or less. The distribution of follow-up time across the study population is presented in supplementary S2 Table.

From birth to 10 years of age, the overall incidence rate of NDD/D was 4.8 cases per 1,000 person-years (95% CI 4.1, 5.4) in Métis children and 4.3 cases per 1,000 person-years (95% CI 4.0, 4.6) in non-Métis children. When analyzed by individual years of life (Table 3), adjusted age-specific incidence rates of NDD/D gradually increased with age in both groups. However, a statistically significant difference was observed only at 2 years of age, where Métis children had a higher adjusted incidence rate (5.5 cases per 1,000 person-years, 95% CI 3.7, 7.3) compared to 2.8 cases per 1,000 person-years (95% CI 2.1, 3.4) in non-Métis children, with a rate difference of 2.7 (95% CI 0.8, 4.6). At all other ages, differences in incidence rates between Métis and non-Métis children were not statistically significant.

**Table 3:** Adjusted age-specific incidence rates of NDD/D in Métis and non-Métis children in Métis and non-Métis children by age.

Age (years)	Métis aIR (95% CI)^1^	Non-Métis aIR (95% CI)^a^	Difference RD (95% CI)
1	3.4 (2.0, 4.7)	2.9 (2.2, 3.6)	0.5 (−1.0, 2.0)
2	5.5 (3.7, 7.3)	2.8 (2.1, 3.4)	**2.7 (0.8, 4.7)**
3	3.1 (1.8, 4.4)	4.3 (3.4, 5.2)	−1.2 (−2.8, 0.4)
4	4.3 (2.7, 5.9)	4.1 (3.3, 5.0)	0.2 (−1.6, 2.0)
5	4.6 (2.7, 6.5)	4.1 (3.2, 5.0)	0.5 (−1.6, 2.6)
6	4.7 (2.4, 7.0)	4.1 (3.2, 5.1)	0.5 (−2.0, 3.1)
7	5.5 (3.3, 7.8)	5.1 (4.1, 6.2)	0.4 (−2.1, 2.9)
8	6.5 (4.2, 8.8)	5.9 (4.8, 7.1)	0.6 (−2.0, 3.2)
9	6.0 (3.1, 8.9)	5.6 (4.4, 6.8)	0.4 (−2.8, 3.6)
10	5.7 (3.1, 8.2)	6.5 (5.0, 8.0)	−0.8 (−3.8, 2.3)

aIR = adjusted incidence rate (per 1,000 person-years); CI = confidence interval; RD = rate difference between Métis and non-Métis groups.

^a^ Adjusted for maternal age, area of residence, material and social deprivation, smoking/substance use during pregnancy, at least one pre-existing medical condition/high pre-pregnancy weight, at least one pregnancy-related condition, prenatal care, delivery mode, infant sex, preterm birth, small for gestational age (non-PTB), large for gestational age, and any congenital anomaly.

### Maternal and neonatal factors associated with NDD/D incidence in Métis children

Several maternal and neonatal factors were associated with an increased incidence of NDD/D among Métis children (Table 4). Among Métis children, the incidence rate of NDD/D was significantly higher in those born to mothers younger than 20 years of age (aIRR 2.1, 95% CI 1.2, 3.7), and in those born to mothers aged 35 years or older (aIRR 1.7, 95% CI 1.1, 2.6), compared to children born to mothers aged 20–34 years. Additionally, maternal pre-pregnancy weight exceeding 91 kg was associated with a higher incidence rate of NDD/D (aIRR 1.8, 95% CI 1.2, 2.8). Maternal smoking or substance use during pregnancy were not significantly associated with an increased incidence of NDD/D (aIRR 1.8, 95% CI: 1.0, 3.1).

**Table 4:** Association between maternal and neonatal characteristics and NDD/D incidence among Métis and non-Métis children.

Characteristic	aIRR (95% CI) Métis	aIRR (95% CI) non-Métis
Maternal age (ref: 20–34)		
< 20	**2.1 (1.2, 3.7)**	**1.8 (1.2, 2.7)**
≥ 35	**1.7 (1.1, 2.6)**	0.9 (0.8, 1.2)
Urban residence at delivery (ref: rural)	1.4 (1.0, 2.0)	
Material deprivation index (ref: Q_1_-least deprived)		
Q_2_	1.5 (0.8, 2.7)	1.0 (0.8, 1.3)
Q_3_	0.9 (0.5, 1.6)	1.0 (0.8, 1.3)
Q_4_	0.8 (0.4, 1.5)	0.9 (0.7, 1.2)
Q_5_ (most deprived)	1.0 (0.5, 1.8)	0.9 (0.7, 1.2)
Social deprivation index (ref: Q_1_-least deprived)		
Q_2_	1.1 (0.6, 2.0)	1.1 (0.8, 1.4)
Q_3_	0.7 (0.4, 1.3)	1.0 (0.7, 1.3)
Q_4_	1.1 (0.6, 1.9)	1.3 (1.0, 1.7)
Q_5_ (most deprived)	1.0 (0.6, 1.9)	**1.4 (1.1, 1.9)**
High pre-pregnancy weight (≥91 kg) (ref: No)	**1.8 (1.2, 2.8)**	1.3 (1.0, 1.7)
Smoke, alcohol, or substance use (ref: no)	1.8 (1.0, 3.1)	**1.9 (1.3, 3.0)**
Any pre-existing medical condition (ref: no)	1.5 (1.0, 2.3)	1.3 (1.0, 1.7)
Any pregnancy-related condition	0.8 (0.5, 1.2)	1.2 (1.0, 1.5)
Prenatal care (ref: Adequate)		
Intermediate	0.9 (0.6, 1.4)	0.8 (0.7, 1.0)
Intensive	2.0 (0.8, 5.1)	0.8 (0.4, 1.6)
Inadequate	0.8 (0.5, 1.5)	0.9 (0.7, 1.2)
No care	0.3 (0.1, 1.1)	0.5 (0.3, 0.8)
Delivery mode (ref: Spontaneous vaginal)		
Vacuum/Forceps	1.6 (1.0, 2.6)	1.3 (1.0, 1.7)
Cesarean	1.2 (0.8, 1.8)	**1.4 (1.2, 1.7)**
Male sex at birth (ref: female)	**1.9 (1.4, 2.7)**	**2.2 (1.9, 2.6)**
Congenital anomaly (ref: No)	**4.5 (2.2, 9.2)**	**5.3 (3.3, 8.4)**
Preterm birth (ref: No)	**2.9 (1.7, 4.9)**	1.3 (0.9, 1.7)
Small for gestational age (ref: No)	1.5 (0.9, 2.6)	1.4 (1.0, 1.8)
Large for gestational age (ref: No)	1.0 (0.6, 1.7)	1.1 (0.9, 1.5)

IRR = incidence rate ratio; CI = confidence interval;

^a^ aIRR = adjusted IRR for all variables listed in this table in a multivariable model.

**Bolded** IRRs/aIRRs indicate 95% CIs excluding 1.0.

Among neonatal characteristics, male Métis infants had nearly twice the incidence rate of NDD/D compared to females (aIRR 1.9, 95% CI 1.4, 2.7). Preterm birth was associated with a nearly threefold increase in NDD/D incidence (aIRR 2.9, 95% CI 1.7, 4.9). The strongest association was observed among Métis infants with congenital anomalies, who had an incidence rate of NDD/D 4.5 times higher (aIRR 4.5, 95% CI 2.2, 9.1) than Métis infants without congenital anomalies. Crude and adjusted associations in both Métis and non-Métis cohorts are presented in supplementary S3 Table.

## Discussion

This is the first population-based study in Canada to examine the epidemiology and associated maternal and neonatal factors of NDD/D among Métis and non-Métis children. Our findings indicate that overall prevalence and incidence rates of NDD/D were similar between the two groups after adjusting for maternal and neonatal factors. Métis children were more likely to be diagnosed at age 2. Additionally, younger (<20 years) or older (≥35 years) maternal age, high pre-pregnancy weight, male sex, preterm birth, and congenital anomalies were significantly associated with increased NDD/D incidence among Métis children.

The finding that Métis children had similar overall rates of NDD/D compared to non-Métis children contrasts with previous research examining disability burdens among Métis populations. Data from the Aboriginal Peoples Survey, indicate that Métis individuals aged 15 years and older reported higher rates of learning and developmental disabilities than non-Indigenous individuals [44]. These self-reported disabilities suggest that Métis individuals may experience a disproportionate burden of neurodevelopmental conditions, yet our study does not reflect such disparities. The discrepancy could be attributed to methodological differences, including reliance on administrative health data rather than self-reported information. Additionally, structural barriers affecting Métis families –such as challenges in accessing specialized diagnostic services– may contribute to lower recorded prevalence rates in health system datasets.

Our findings also contrast with previous research on neurodevelopmental health of Indigenous children in Alberta. A study by Burstyn et al. [11] found lower rates of ASD among Indigenous children in Alberta based on administrative health data, though the study aggregated First Nations, Inuit, and Métis children, making direct comparisons difficult. Given the evidence suggesting that Indigenous children may be underdiagnosed due to limited access to healthcare services and diagnostic tools, the lower ASD rates observed in Burstyn et al. study [11] may reflect systemic underrepresentation rather than a true epidemiological trend. This highlights the need for Métis-specific research to disentangle the unique characteristics of neurodevelopmental health of Métis children from those of other Indigenous groups.

When considering Indigenous children more broadly across Canada, similar trends emerge. Research with First Nations children [45] suggests they may be less likely to receive an autism or NDD/D diagnosis due to systemic healthcare inequities, cultural barriers, and diagnostic tools that do not reflect Indigenous perspectives on child development.

Internationally, similar patterns have been documented. Tupou et al. [46] reported that Māori children in Aotearoa/New Zealand had slightly higher ASD prevalence than their non-Māori peers, though cultural perceptions of disability and access barriers to diagnosis likely contribute to underrepresentation in official health records and administrative health datasets. A scoping review of studies on ASD among Indigenous Australians found that prevalence was comparable to non-Indigenous Australians but emphasized that misdiagnosis and lack of access to services likely contributed to diagnostic disparities [47].

Taken together, this body of evidence suggests that the similar rates of NDD/D observed between Métis and non-Métis children in our study may reflect systemic diagnostic inequities rather than true epidemiological similarities.

In Canada, NDD/D diagnoses are often triggered by parental concern, referrals from educators or primary care providers, or challenges encountered in school settings. These diagnostic pathways can be influenced by long wait times, geographic remoteness, systemic bias, and limited access to culturally appropriate services, all of which may disproportionately affect Métis and other Indigenous families [17,48,49]. As a result, administrative health records may underrepresent the true burden of NDD/D in these populations.

Age at diagnosis may also contribute to observed differences in prevalence across studies. Many NDD/D conditions, including ASD, are not diagnosed until later childhood due to psychometric limitations of early screening tools and delayed access to specialists [50]. While our finding that Métis children had a higher incidence of NDD/D at age 2 suggests that some early screening mechanisms may be in place, disparities in diagnosis likely persist. Because healthcare access significantly influences the visibility of NDD/D in administrative health data, reliance on these sources may further underestimate true prevalence, particularly in underserved populations [51].

Indigenous families in Canada face significant barriers to accessing neurodevelopmental assessment and diagnostic services, including long wait times, lack of culturally sensitive care, and limited awareness about available support [17]. As a result, the true burden of NDD/D among Métis children may be underestimated in administrative health records.

The development of NDD/D is multifactorial, involving genetic, environmental, and social factors [28]. When exploring maternal and neonatal factors associated with NDD/D incidence in Métis children, maternal age under 20 and over 35, high pre-pregnancy weight, male sex, preterm birth, and congenital anomalies, emerged as key factors. Many of these characteristics are also known to influence brain development in other populations [30,32]. It is likely that these factors act synergistically in increasing NDD/D risk. For example, Raoufi et al. [8] found that children from lower SES backgrounds with housing instability and healthcare access barriers had higher severity of NDD/D symptoms. Similarly, Amjad et al. [52] reported that adolescent mothers from lower SES and rural areas were more likely to smoke, use substances, and experience adverse birth outcomes such as preterm birth, all of which are associated with increased NDD/D risk. These findings suggest that the intersection of socioeconomic deprivation and maternal health disparities may amplify NDD/D risk among Métis children.

The social determinants of health, deeply shaped by colonialism, are among the key contributors to Indigenous children’s health disparities [16,53]. Colonial policies have disrupted Métis families and communities through forced displacement, loss of cultural identity, and systemic inequities in healthcare and education [15]. Research has shown that the historical overemphasis on Fetal Alcohol Spectrum Disorder (FASD) in Indigenous health research has contributed to a narrow and often stigmatizing lens through which Indigenous children’s neurodevelopmental health is understood, potentially limiting attention to other conditions such as ASD or intellectual disabilities [54]. Addressing these systemic issues is crucial when designing programs and policies to support Métis children and families.

The findings from this study have important clinical and policy implications. The lack of statistically significant differences in overall NDD/D prevalence between Métis and non-Métis children after adjusting for maternal and neonatal factors suggests that improving maternal health, prenatal care, and neonatal outcomes may help promote equitable developmental trajectories. While we observed a higher incidence rate of NDD/D at age 2 among Métis children, this may reflect earlier screening or detection mechanisms already in place rather than a true difference in underlying risk. Future research is needed to determine whether this early diagnosis is driven by differences in access to screening programs or a true reflection of incidence at earlier ages. It is also important to explore the developmental consequences and service trajectories following early diagnosis –whether early identification leads to improved long-term outcomes, or weather disparities emerge later in childhood. Additionally, given the strong associations between preterm birth, congenital anomalies, and NDD/D, targeted interventions for at-risk neonates may help improve early developmental outcomes.

Clinicians should be aware of the sociocultural and structural barriers that influence neurodevelopmental health of Métis children. The development of Métis-led psychosocial interventions that support Métis women during pregnancy and Métis children postnatally could play a critical role in promoting better neurodevelopmental trajectories. Furthermore, future research should be conducted in partnership with Métis communities to further examine the relationship between social inequities and NDD/D. Integrating Métis ways of knowing into research and intervention design is necessary for creating culturally relevant alternatives.

### Strengths and limitations

A major strength of this study is its collaborative approach with the MNA. This research applies epidemiological methods in partnership with Métis people, aiming to describe disparities while upholding Indigenous data sovereignty and ensuring findings are relevant to Métis communities. Additionally, this study takes a functional approach to neurodevelopmental health, which focuses on developmental and behavioral characteristics rather than rigid diagnostic categories [4,6]. This perspective is particularly valuable in shaping policy and service organization, as it highlights specific functional needs rather than imposing potentially stigmatizing labels. By emphasizing functional outcomes, this approach facilitates more inclusive, responsive, and accessible interventions for Métis children with neurodevelopmental challenges.

The study also has limitations. One important limitation is the composition of the non-Métis comparison group, which may have included children from other Indigenous groups (First Nations and Inuit) as well as racialized populations. This may have influenced our findings, particularly if these groups experience similar structural barriers to diagnosis and care. According to the 2021 Canadian Census, there are approximately 41,000 First Nations and Inuit children aged 14 and under living in Alberta [19], representing a sizable proportion of the pediatric population and highlighting the potential for misclassification bias in comparative analyses. Their inclusion in the non-Métis group could bias estimates towards the null, potentially obscuring differences in NDD/D prevalence that may exist between Métis and non-Indigenous children. Additionally, the study population is limited to MNA citizens and the findings may not be fully representative of all Métis children in Alberta, particularly those who may not be registered or who belong to other Métis organizations.

A major limitation inherent in any retrospective study is the reliance on administrative health data, which lacks important clinical data and relies on predefined categorizations. The inability to account for individual-level clinical and social factors introduces the potential for residual confounding, where unmeasured or imprecisely measured variables may still have a significant influence on observed outcomes. Additionally, administrative datasets often reflect healthcare access rather than true prevalence, meaning underdiagnosis and differential healthcare engagement could affect the results. Finally, the study period (2006–2016) reflects the timeframe agreed upon in the research agreement between the MNA, the administrative health data custodians and the investigators. While this period allows for a robust analysis of neurodevelopmental trends over a decade, it also means that more recent changes in diagnostic practices, service availability, and healthcare policies are not captured. Advances in screening protocols, shifts in diagnostic criteria, and increased awareness of neurodevelopmental conditions in Indigenous communities may all impact the generalizability of findings to present-day contexts. Despite these limitations, this study provides critical insights into Métis children’s neurodevelopmental health and highlights the need for continued research using community-driven methodologies and more comprehensive data sources.

## Conclusions

Métis and non-Métis children had similar overall prevalence and incidence rates of NDD/D after adjusting for maternal and neonatal factors. However, Métis children had a higher incidence rate of NDD/D at age 2, suggesting potential differences in early diagnosis, access to care, or underlying risk factors. Among Métis children, maternal age younger than 20 or older than 35 years, high pre-pregnancy weight, male sex, preterm birth, and congenital anomalies were significantly associated with increased NDD/D incidence. Applying a functional classification approach to NDD/D offers a broader understanding of impairments beyond diagnostic labels and provides a useful framework for research, service planning, and policy development. These findings emphasize the need for targeted and culturally responsive early screening and interventions that address social and perinatal risk factors. Supporting Métis children’s neurodevelopmental health requires addressing systemic inequities and the broader social determinants of health through community-driven approaches.

## Supporting information

S1 Table ICD-9 and ICD-10-CA codes for neurodevelopmental disorders and disabilities. (DOCX)

S2 Table Frequency and percentage of children by follow-up years of age in the study cohort (2006–2016 singleton live births in Alberta). (DOCX)

S3 Table Crude and adjusted associations between maternal and neonatal characteristics and the incidence of NDD/D among Métis and non-Métis children. (DOCX)

S4 File Checklist. Inclusivity in global research questionnaire. (DOCX)

## References

[1] American Psychiatric Association. Diagnostic and statistical manual of mental disorders. 5th ed. Arlington (VA): American Psychiatric Association. 2013.

[2] World Health Organization. International statistical classification of diseases and related health problems. 10th ed. Geneva: World Health Organization. 1992.

[3] World Health Organization. International Classification of Functioning, Disability and Health (ICF). n.d. https://www.who.int/standards/classifications/international-classification-of-functioning-disability-and-health

[4] Miller AR, Mâsse LC, Shen J, Schiariti V, Roxborough L. Diagnostic status, functional status and complexity among Canadian children with neurodevelopmental disorders and disabilities: a population-based study. Disabil Rehabil. 2013;35(6):468–78. doi: 10.3109/09638288.2012.699580 22794277

[5] Arim RG, Kohen DE, Brehaut JC, Guèvremont A, Garner RE, Miller AR, et al. Developing a non-categorical measure of child health using administrative data. Health Rep. 2015;26(2):9–16. 25692939

[6] Arim RG, Miller AR, Guèvremont A, Lach LM, Brehaut JC, Kohen DE. Children with neurodevelopmental disorders and disabilities: a population-based study of healthcare service utilization using administrative data. Dev Med Child Neurol. 2017;59(12):1284–90. doi: 10.1111/dmcn.13557 28905997

[7] Bush KL, Tassé MJ. Employment and choice-making for adults with intellectual disability, autism, and down syndrome. Res Dev Disabil. 2017;65:23–34. doi: 10.1016/j.ridd.2017.04.004 28433791

[8] Raouafi S, Achiche S, Raison M. Socioeconomic disparities and difficulties to access to healthcare services among Canadian children with neurodevelopmental disorders and disabilities. Epidemiol Health. 2018;40:e2018010. doi: 10.4178/epih.e2018010 29642656 PMC6004430

[9] Office of Statistics and Information - Demography. 2016 Census of Canada: Aboriginal People. Edmonton: Alberta Government. 2017.

[10] Baydala L, Sherman J, Rasmussen C, Wikman E, Janzen H. ADHD characteristics in Canadian Aboriginal children. J Atten Disord. 2006;9(4):642–7. doi: 10.1177/1087054705284246 16648231

[11] Burstyn I, Sithole F, Zwaigenbaum L. Autism spectrum disorders, maternal characteristics and obstetric complications among singletons born in Alberta, Canada. Chronic Dis Can. 2010;30(4):125–34. 20946713

[12] Chen A, Dyck Holzinger S, Oskoui M, Shevell M, Canadian Cerebral Palsy Registry. Cerebral palsy in Canadian Indigenous children. Dev Med Child Neurol. 2021;63(5):614–22. 33314061 10.1111/dmcn.14776

[13] Métis National Council. Métis Perspectives on Disability Research and the United Nations Convention on the Rights of Persons with Disabilities. 2025. https://www.metisnation.ca/uploads/documents/2025%2004%20Metis%20Perspectives%20on%20Disbility%20Research.pdf

[14] Hus Y. Frozen in Time, a Focused Review of Autism Prevalence in Canadian Indigenous Communities. Neuropsychiatr Dis Treat. 2023;19:2451–68. doi: 10.2147/NDT.S439450 38029046 PMC10658944

[15] Matheson K, Seymour A, Landry J, Ventura K, Arsenault E, Anisman H. Canada’s Colonial Genocide of Indigenous Peoples: A Review of the Psychosocial and Neurobiological Processes Linking Trauma and Intergenerational Outcomes. Int J Environ Res Public Health. 2022;19(11):6455. doi: 10.3390/ijerph19116455 35682038 PMC9179992

[16] Greenwood ML, de Leeuw SN. Social determinants of health and the future well-being of Aboriginal children in Canada. Paediatr Child Health. 2012;17(7):381–4. 23904782 PMC3448539

[17] Gerlach AJ, Matthiesen A, Moola FJ, Watts J. Autism and autism services with Indigenous families and children in the settler-colonial context of Canada: A critical scoping review. Can J Disabil Stud. 2022;11(2):1–39.

[18] Lau SC, Czuczman NM, Dennett L, Hicks M, Ospina MB. Systematic Review and Meta-Analysis: Prevalence of Neurodevelopmental Disorders Among Indigenous Children. JAACAP Open. 2024;3(3):406–20. doi: 10.1016/j.jaacop.2024.02.007 40922776 PMC12414266

[19] Statistics Canada. Focus on Geography Series, 2021 Census - Alberta. 2022. https://www12.statcan.gc.ca/census-recensement/2021/as-sa/fogs-spg/page.cfm?lang=E&topic=8&dguid=2021A000248

[20] Statistics Canada. The Daily — Indigenous population continues to grow and is much younger than the non-Indigenous population, although the pace of growth has slowed. 2022. https://www150.statcan.gc.ca/n1/daily-quotidien/220921/dq220921a-eng.htm

[21] Ospina MB, Sanni OB, Serrano-Lomelin J, James A, Bradburn K, Bartel R, et al. Ehawawisit: Sociodemographic and Clinical Characteristics and Perinatal Outcomes of Métis Pregnancies in Alberta, Canada. J Obstet Gynaecol Can. 2025;47(9):103044. doi: 10.1016/j.jogc.2025.103044 40675374

[22] Statistics Canada. An update on the socio-economic gaps between Indigenous Peoples and the non-Indigenous population in Canada: Highlights from the 2021 Census. 2023. https://www.sac-isc.gc.ca/eng/1690909773300/1690909797208

[23] Statistics Canada. Indigenous identity population by gender and age: Canada, provinces and territories, census metropolitan areas and census agglomerations. 2023. https://open.canada.ca/data/en/dataset/46f1d5c9-0df8-45e9-b4a1-1e7da28b1560/resource/eb73f790-8b55-4804-b05a-6c3dc5e301f8

[24] Canadian Institute for Health Information. The Canadian enhancement of ICD-10. Ottawa, ON: Canadian Institute for Health Information. 2001.

[25] Canadian Institute for Health Information. Canadian Classification of Health Interventions - Tabular List. Ottawa (ON): CIHI. 2012.

[26] World Health Organization. International Statistical Classification of Diseases, Injuries, and Causes of Death, Ninth Revision. Genève: World Health Organization. 1979.

[27] Hardie JH, Landale NS. Profiles of Risk: Maternal Health, Socioeconomic Status, and Child Health. J Marriage Fam. 2013;75(3):651–66. doi: 10.1111/jomf.12021 23794751 PMC3685849

[28] De Felice A, Ricceri L, Venerosi A, Chiarotti F, Calamandrei G. Multifactorial Origin of Neurodevelopmental Disorders: Approaches to Understanding Complex Etiologies. Toxics. 2015;3(1):89–129. doi: 10.3390/toxics3010089 29056653 PMC5634696

[29] Ehrenstein V, Pedersen L, Grijota M, Nielsen GL, Rothman KJ, Sørensen HT. Association of Apgar score at five minutes with long-term neurologic disability and cognitive function in a prevalence study of Danish conscripts. BMC Pregnancy Childbirth. 2009;9:14. doi: 10.1186/1471-2393-9-14 19341459 PMC2670812

[30] Kundakovic M, Jaric I. The Epigenetic Link between Prenatal Adverse Environments and Neurodevelopmental Disorders. Genes (Basel). 2017;8(3):104. doi: 10.3390/genes8030104 28335457 PMC5368708

[31] Fernández de Gamarra-Oca L, Ojeda N, Gómez-Gastiasoro A, Peña J, Ibarretxe-Bilbao N, García-Guerrero MA, et al. Long-Term Neurodevelopmental Outcomes after Moderate and Late Preterm Birth: A Systematic Review. J Pediatr. 2021;237:168-176.e11. doi: 10.1016/j.jpeds.2021.06.004 34171360

[32] Wehby GL, Prater K, McCarthy AM, Castilla EE, Murray JC. The Impact of Maternal Smoking during Pregnancy on Early Child Neurodevelopment. J Hum Cap. 2011;5(2):207–54. doi: 10.1086/660885 22272363 PMC3262676

[33] Zhang T, Sidorchuk A, Sevilla-Cermeño L, Vilaplana-Pérez A, Chang Z, Larsson H, et al. Association of Cesarean Delivery With Risk of Neurodevelopmental and Psychiatric Disorders in the Offspring: A Systematic Review and Meta-analysis. JAMA Netw Open. 2019;2(8):e1910236. doi: 10.1001/jamanetworkopen.2019.10236 31461150 PMC6716295

[34] Public Health Agency of Canada. Perinatal health indicators for Canada 2013: A report of the Canadian Perinatal Surveillance System. Ottawa. 2013. http://www.phac-aspc.gc.ca/rhs-ssg/phi-isp-2013-eng.php

[35] Rowe S, Karkhaneh Z, MacDonald I, Chambers T, Amjad S, Osornio-Vargas A, et al. Systematic review of the measurement properties of indices of prenatal care utilization. BMC Pregnancy Childbirth. 2020;20(1):171. doi: 10.1186/s12884-020-2822-5 32183724 PMC7079477

[36] Alberta Health Services, Alberta Health. 2018. https://open.alberta.ca/dataset/a14b50c9-94b2-4024-8ee5-c13fb70abb4a/resource/70fd0f2c-5a7c-45a3-bdaa-e1b4f4c5d9a4/download/Official-Standard-Geographic-Area-Document.pdf

[37] Pampalon R, Hamel D, Gamache P, Philibert MD, Raymond G, Simpson A. An area-based material and social deprivation index for public health in Québec and Canada. Can J Public Health. 2012;103(8 Suppl 2):S17-22. doi: 10.1007/BF03403824 23618066 PMC6973787

[38] Kramer MS, Platt RW, Wen SW, Joseph KS, Allen A, Abrahamowicz M, et al. A new and improved population-based Canadian reference for birth weight for gestational age. Pediatrics. 2001;108(2):E35. doi: 10.1542/peds.108.2.e35 11483845

[39] El Emam K, Dankar FK. Protecting privacy using k-anonymity. J Am Med Inform Assoc. 2008;15(5):627–37. doi: 10.1197/jamia.M2716 18579830 PMC2528029

[40] McGrath O, Burke K. Binomial confidence intervals for rare events: Importance of defining margin of error relative to magnitude of proportion. Am Statist. 2024;78(4):437–49.

[41] Benchimol EI, Smeeth L, Guttmann A, Harron K, Moher D, Petersen I, et al. The REporting of studies Conducted using Observational Routinely-collected health Data (RECORD) statement. PLoS Med. 2015;12(10):e1001885. doi: 10.1371/journal.pmed.1001885 26440803 PMC4595218

[42] Huria T, Palmer SC, Pitama S, Beckert L, Lacey C, Ewen S, et al. Consolidated criteria for strengthening reporting of health research involving indigenous peoples: the CONSIDER statement. BMC Med Res Methodol. 2019;19(1):173. doi: 10.1186/s12874-019-0815-8 31399058 PMC6688310

[43] National Aboriginal Health Organization Métis Centre. Principles of Ethical Métis Research. 2017. https://achh.ca/wp-content/uploads/2018/07/Guide_Ethics_NAHOMetisCentre.pdf

[44] Statistics Canada. Indigenous People with Disabilities in Canada: First Nations People Living Off Reserve, Métis and Inuit Aged 15 Years and Older. n.d. https://www150.statcan.gc.ca/n1/pub/89-653-x/89-653-x2019005-eng.htm

[45] Lindblom A. Under-detection of autism among First Nations children in British Columbia, Canada. Disabil Soc. 2014;29(8):1248–59.

[46] Tupou J, Curtis S, Taare-Smith D, Glasgow A, Waddington H. Māori and autism: A scoping review. Autism. 2021;25(7):1844–58. doi: 10.1177/13623613211018649 34088216

[47] Bailey B, Arciuli J. Indigenous Australians with autism: A scoping review. Autism. 2020;24(5):1031–46. doi: 10.1177/1362361319894829 31928063 PMC7309356

[48] Horrill T, McMillan DE, Schultz ASH, Thompson G. Understanding access to healthcare among Indigenous peoples: A comparative analysis of biomedical and postcolonial perspectives. Nurs Inq. 2018;25(3):e12237. doi: 10.1111/nin.12237 29575412 PMC6055798

[49] Ouellette-Kuntz H, Coo H, Yu CT, Chudley AE, Noonan A, Breitenbach M. Prevalence of pervasive developmental disorders in two Canadian provinces. J Policy Pract Intellect Disabil. 2006;3(3):164–72.

[50] Villagomez AN, Muñoz FM, Peterson RL, Colbert AM, Gladstone M, MacDonald B, et al. Neurodevelopmental delay: Case definition & guidelines for data collection, analysis, and presentation of immunization safety data. Vaccine. 2019;37(52):7623–41. doi: 10.1016/j.vaccine.2019.05.027 31783983 PMC6899448

[51] Smylie J, Firestone M. Back to the basics: Identifying and addressing underlying challenges in achieving high quality and relevant health statistics for indigenous populations in Canada. Stat J IAOS. 2015;31(1):67–87. doi: 10.3233/SJI-150864 26793283 PMC4716822

[52] Amjad S, Chandra S, Osornio-Vargas A, Voaklander D, Ospina MB. Maternal Area of Residence, Socioeconomic Status, and Risk of Adverse Maternal and Birth Outcomes in Adolescent Mothers. J Obstet Gynaecol Can. 2019;41(12):1752–9. doi: 10.1016/j.jogc.2019.02.126 31047831

[53] King M, Smith A, Gracey M. Indigenous health part 2: the underlying causes of the health gap. Lancet. 2009;374(9683):76–85. doi: 10.1016/S0140-6736(09)60827-8 19577696

[54] Di Pietro NC, Illes J. Disparities in Canadian indigenous health research on neurodevelopmental disorders. J Dev Behav Pediatr. 2014;35(1):74–81. doi: 10.1097/DBP.0000000000000002 24356498

