# Supplementary material for: Incidence and prevalence of neurodevelopmental disorders and disabilities among métis children in Alberta, Canada: A retrospective birth cohort study
Source: PLoS One. 2025 Oct 3;20(10):e0333699. doi: 10.1371/journal.pone.0333699 (PMC12494283; doi:10.1371/journal.pone.0333699)
Supplement: S1 Table — (DOCX) [file pone.0333699.s001.docx]

**S1 Table**. ICD-9 and ICD-10-CA codes for neurodevelopmental disorders and disabilities

| **Domain** | **Health outcome** | **ICD-9** | **ICD-10** |
| --- | --- | --- | --- |
| Motor functioning | Infantile cerebral palsy | 343 | G80.9 |
|  | Spina bifida | 741 | Q05.* |
|  | Lack of coordination | 781.3 | R27* |
|  | Muscular dystrophies and other myopathies | 359 | G71* |
| Speech  language  communication | Speech disturbances | 784.5 | R47.8 |
|  | Problems with voice production | V41.4 | R47.89 |
|  | Aphasia | 784.3 | R47.01 |
|  | Developmental speech or language disorder | 315.3 | F80.* |
| Learning  Cognition | Developmental disorder of scholastic skills | 315.9 | F81.9 |
|  | Mental and behavioral problems with learning | V40.0 | F81.9^a^ |
|  | Fetal alcohol syndrome | 760.71 | Q86.0 / P04.3 |
|  | Reading disorder | 315 | F81.0 |
|  | Other symbolic disfunction | 784.6 | R48.9 |
|  | Signs and symptoms involving cognition | 799.5 | R41.8 |
|  | Attention deficit hyperactivity disorder | 314.0 | F90.* |
|  | Unspecified intellectual disabilities | 319 | F79 |
|  | Down syndrome | 758 | Q90 |
| Reciprocal social interaction | Largely autism and its variants | 299.* | F84.* |
| Sensory | Deafness (conductive hearing loss) | 389 | H91.9 |
| impairments | Blindness and low vision | 369.* | H54.0 / H54.7 |
|  | Problems with special senses and other special functions | V41 |  |
| Neurophysiological | Epilepsy and recurrent seizures | 345.* | G40* |
|  | Tourette's disorder | 307.2 | F95.2 |
|  |  |  |  |

^a^ The ICD-10 code F81.9 of mental and behavioral problems with learning was included under F81.9 of developmental disorder of scholastic skills to avoid duplicates.
