# Supplementary material for: Incidence and prevalence of neurodevelopmental disorders and disabilities among métis children in Alberta, Canada: A retrospective birth cohort study
Source: PLoS One. 2025 Oct 3;20(10):e0333699. doi: 10.1371/journal.pone.0333699 (PMC12494283; doi:10.1371/journal.pone.0333699)
Supplement: S2 Table — (DOCX) [file pone.0333699.s002.docx]

**S2 Table**. Frequency and percentage of children by follow-up years of age* in the study cohort (2006-2016 singleton live births in Alberta).

| **Follow-up time (Years)*** | **Frequency (N)** | **Percent (%)** |
| --- | --- | --- |
| 10 | 12,830 | 32.9 |
| 9 | 3,700 | 9.5 |
| 8 | 3,828 | 9.8 |
| 7 | 3,875 | 9.9 |
| 6 | 4,049 | 10.4 |
| 5 | 4,211 | 10.8 |
| 4 | 4,371 | 11.2 |
| 3 | 1,300 | 3.3 |
| 2 | 454 | 1.2 |
| ≥1 | 340 | 0.9 |
| **Total** | **38,958** | **100.0** |

*The follow-up period of each child ran from their date of birth (between April 1, 2006, and March 31, 2016) until they turned ten years old, or the end of the study period (March 31, 2019), or when they died or moved out of the province
