# Supplementary material for: Incidence and prevalence of neurodevelopmental disorders and disabilities among métis children in Alberta, Canada: A retrospective birth cohort study
Source: PLoS One. 2025 Oct 3;20(10):e0333699. doi: 10.1371/journal.pone.0333699 (PMC12494283; doi:10.1371/journal.pone.0333699)
Supplement: S3 Table — (DOCX) [file pone.0333699.s003.docx]

**S3 Table**. Crude and adjusted associations between maternal and neonatal characteristics and the incidence of NDD/D among Métis and non-Métis children.

|  |  | |  | |  | |  |  |
| --- | --- | --- | --- | --- | --- | --- | --- | --- |
| **Characteristic** | **Crude IRR**  **(95% CI)** | **aIRR**  **(95% CI)** | | **Crude IRR**  **(95% CI)** | | **aIRR**  **(95% CI)** | | |
| Maternal age (ref: 20-34) |  |  | |  | |  | | |
| < 20 | **1.6 (1.1, 2.3)** | **2.1 (1.2, 3.7)** | | **1.5 (1.1, 2.1)** | | **1.8 (1.2, 2.7)** | | |
| ≥ 35 | **1.6 (1.1, 2.3)** | **1.7 (1.1, 2.6)** | | 1.0 (0.8, 1.2) | | 0.9 (0.8, 1.2) | | |
| Urban residence at delivery (ref: rural) | **1.6 (1.2, 2.1)** | 1.4 (1.0, 2.0) | | **1.2 (1.1, 1.5)** | |  | | |
| Material deprivation (ref: Q_1_-least deprived) |  |  | |  | |  | | |
| Q_2_ | 1.4 (0.8, 2.2) | 1.5 (0.8, 2.7) | | 1.0 (0.8, 1.2) | | 1.0 (0.8, 1.3) | | |
| Q_3_ | 1.0 (0.6, 1.7) | 0.9 (0.5, 1.6) | | 1.0 (0.8, 1.2) | | 1.0 (0.8, 1.3) | | |
| Q_4_ | 0.9 (0.6, 1.6) | 0.8 (0.4, 1.5) | | 1.0 (0.7, 1.2) | | 0.9 (0.7, 1.2) | | |
| Q_5_ (most deprived) | 1.0 (0.6, 1.6) | 1.0 (0.5, 1.8) | | 0.9 (0.7, 1.2) | | 0.9 (0.7, 1.2) | | |
| Social deprivation (ref: Q_1_-least deprived) |  |  | |  | |  | | |
| Q_2_ | 1.1 (0.7, 1.9) | 1.1 (0.6, 2.0) | | 1.1 (0.8, 1.4) | | 1.1 (0.8, 1.4) | | |
| Q_3_ | 0.9 (0.6, 1.5) | 0.7 (0.4, 1.3) | | 1.0 (0.7, 1.3) | | 1.0 (0.7, 1.3) | | |
| Q_4_ | 1.2 (0.8, 1.9) | 1.1 (0.6, 1.9) | | 1.3 (1.0, 1.6) | | 1.3 (1.0, 1.7) | | |
| Q_5_ (most deprived) | 1.2 (0.7, 1.9) | 1.0 (0.6, 1.9) | | **1.5 (1.1, 1.9)** | | **1.4 (1.1, 1.9)** | | |
| Pre-pregnancy weight ≥91 kg (ref: No) | **1.6 (1.1, 2.2)** | **1.8 (1.2, 2.8)** | | **1.4 (1.1, 1.8)** | | 1.3 (1.0, 1.7) | | |
| Smoke/alcohol/substance use (ref: no) | **1.9 (1.1, 3.3)** | 1.8 (1.0, 3.1) | | **2.1 (1.4, 3.2)** | | **1.9 (1.3, 3.0)** | | |
| Any pre-pregnancy medical condition (ref: no) | **1.7 (1.2, 2.4)** | 1.5 (1.0, 2.3) | | **1.4 (1.1, 1.7**) | | 1.3 (1.0, 1.7) | | |
| Any pregnancy-related condition | 1.0 (0.7, 1.4) | 0.8 (0.5, 1.2) | | **1.4 (1.2, 1.7)** | | 1.2 (1.0, 1.5) | | |
| Prenatal care (ref: Adequate) |  |  | |  | |  | | |
| Intermediate | 0.9 (0.7, 1.3) | 0.9 (0.6, 1.4) | | 0.9 (0.7, 1.0) | | 0.8 (0.7, 1.0) | | |
| Intensive | **2.2 (1.1, 4.7)** | 2.0 (0.8, 5.1) | | 1.0 (0.6, 1.7) | | 0.8 (0.4, 1.6) | | |
| Inadequate | 1.0 (0.7, 1.5) | 0.8 (0.5, 1.5) | | 1.0 (0.8, 1.3) | | 0.9 (0.7, 1.2) | | |
| No care | 1.1 (0.5, 2.3) | 0.3 (0.1, 1.1) | | 0.6 (0.4, 1.0) | | 0.5 (0.3, 0.8) | | |
| Delivery mode (ref: Spontaneous vaginal) |  |  | |  | |  | | |
| Vacuum/Forceps | 1.2 (0.8, 1.9) | 1.6 (1.0, 2.6) | | 1.3 (1.0, 1.6) | | 1.3 (1.0, 1.7) | | |
| Cesarean | 1.1 (0.8, 1.5) | 1.2 (0.8, 1.8) | | **1.6 (1.3, 1.8)** | | **1.4 (1.2, 1.7)** | | |
| Male sex at birth (ref: female) | **2.0 (1.6, 2.7)** | **1.9 (1.4, 2.7)** | | **2.3 (2.0, 2.7)** | | **2.2 (1.9, 2.6)** | | |
| Congenital anomaly (ref: No) | **4.2 (2.3, 7.9)** | **4.5 (2.2, 9.2)** | | **5.2 (3.6, 7.7)** | | **5.3 (3.3, 8.4)** | | |
| Preterm birth (ref: No) | **2.6 (1.8, 3.8)** | **2.9 (1.7, 4.9)** | | **1.8 (1.4, 2.3)** | | 1.3 (0.9, 1.7) | | |
| Small for gestational age (ref: No) | 1.1 (0.7, 1.8) | 1.5 (0.9, 2.6) | | **1.4 (1.1, 1.8**) | | 1.4 (1.0, 1.8) | | |
| Large for gestational age (ref: No) | 0.9 (0.6, 1.4) | 1.0 (0.6, 1.7) | | 1.1 (0.9, 1.4) | | 1.1 (0.9, 1.5) | | |

IRR = incidence rate ratio; CI= confidence interval.

^a^aIRR = adjusted IRR. Adjusting for all variables listed in this table in a multivariable model

**Bolded** IRRs/aIRRs indicate 95% CIs excluding 1.0
